# miR-124 targets GATA6 to suppress cholangiocarcinoma cell invasion and metastasis

**DOI:** 10.1186/s12885-017-3166-z

**Published:** 2017-03-07

**Authors:** Feng Tian, Jian Chen, Shuguo Zheng, Dajiang Li, Xin Zhao, Peng Jiang, Jianwei Li, Shuguang Wang

**Affiliations:** Institute of Hepatobiliary Surgery, Southwest Hospital, Third Military Medical University, No. 29 Gaotanyan Street, Shapingba District, Chongqing, 400038 China

**Keywords:** Cholangiocarcinoma, Invasion and metastasis, miR-124, GATA6

## Abstract

**Background:**

Our previous study showed that GATA6 plays important roles in cholangiocarcinoma (CCA) cell invasion and metastasis. However, the regulation mechanism of GATA6 in CCA is not clear. In this study, we studied the potential function of miR-124 in CCA and the mechanism of GATA6 regulation.

**Methods:**

The expression levels of miR-124 and GATA6 in cancerous tissues from 57 CCA patients was detected by RT-PCR and IHC. The impact of miR-124 on GATA6 expression in CCA cells was evaluated using cell transfection, xenotransplantation into nude mice and a luciferase reporter assay.

**Results:**

miR-124 was decreased in 57 cancerous tissue samples compared with 38 matched paracancerous samples. The miR-124 level was inversely associated with lymph node involvement and distant metastasis. miR-124 significantly inhibited invasion and migration of CCA cells in vitro. Furthermore, miR-124 inhibited CCA cell metastasis in nude mice. miR-124 inhibited the luciferase activity of reporter genes containing the wild-type GATA6 3′-UTR, which was abrogated by mutation of the binding site. The protein levels of GATA6 were negatively regulated by miR-124. miR-124 expression was inversely associated with GATA6 in 57 cancerous samples. The miR-124-induced suppression of CCA invasion was abrogated by remedial expression of GATA6. GATA6 expression was decreased by miR-124 overexpression in liver masses from nude mice.

**Conclusions:**

Our data suggested that miR-124 decreases GATA6 expression by targeting its 3′-UTR, which in turn inhibits CCA invasion and metastasis.

**Electronic supplementary material:**

The online version of this article (doi:10.1186/s12885-017-3166-z) contains supplementary material, which is available to authorized users.

## Background

Cholangiocarcinoma (CCA) is a highly malignant cancer with a poor prognosis. Radical resection provides the only option for cure. However, only a small proportion of CCA patients can receive surgery because of the metastatic nature of the disease [1]. Metastasis is a multistep process by which primary tumour cells invade adjacent tissues, enter the bloodstream, survive in circulation, extravasate into the surrounding tissue parenchyma, and finally form clinically detectable metastases [2]. This multistep process is initiated and regulated through the alteration of numerous molecules acting as oncogenes or suppressors. Thus far, a number of altered molecules and the related signalling pathways have been reported [3, 4]. However, the invasion and metastasis of CCA might be regulated by a molecular network that is far from completely understood.

Among the molecules that are altered during CCA progression, our previous study found that GATA6, a member of an evolutionarily conserved family of zinc finger transcription factors, is aberrantly upregulated [5]. In addition, GATA6 promoted CCA invasion and metastasis. These results indicate that GATA6 acts as a potential oncogene in CCA. While the mechanism of GATA6 upregulation in CCA is unclear, the upregulation might be attributed to amplification of promoting factors and inhibition of blocking factors. Kwei et al. [6] reported that amplification of the GATA6 gene might contribute to the aberrant expression of GATA6, which is one amplification promoting factor. However, there are few reports that mention the inhibition of blocking factors of GATA6 in CCA.

MicroRNAs (miRs) are small noncoding RNA oligonucleotides that regulate a large number of genes. They perform the negative regulation through complementary binding to mRNAs 3′-untranslated regions (UTRs). A growing body of evidence suggests that some miRNAs function as onco-miRs or tumour-suppressor miRs by targeting known oncogenes or tumour-suppressor genes [7–10]. Thus, we hypothesized that miRs might participate in negative regulation of GATA6 in CCA. Among the numerous miRNAs, we focused on miR-124 based on the following observations: (1) Recently, mir-124 was reported to be downregulated and to affect metastasis in several types of cancer, including hepatocellular carcinoma, pancreatic cancer, breast cancer, prostate cancer, glioma and lung cancer [11–16]. However, its role in CCA is uncertain. (2) Bioinformatics analysis has shown that the 3′-UTR of GATA6 gene contains a potential miR-124 binding site. (3) Our preliminary experiments indicated that the level of miR-124 is negatively associated with GATA6 in a CCA cell line, QBC939. Thus, we postulate that miR-124 might participate in the regulation of GATA6 in CCA.

In the present study, the expression profile of miR-124 in CCA samples was investigated. The role of miR-124 on CCA cell migration, invasion and metastasis was also investigated. Finally, we examined the mechanism of miR-124 in regulating GATA6 in CCA cells. Based on the present study, we may abandon the mechanism of CCA metastasis. In addition, miR-124 may potentially be a new prognostic indicator and molecular target for treatment of CCA.

## Methods

### Clinical samples

In total, 57 frozen cancerous samples from CCA patients undergoing surgery from 2005 to 2010 at our department were collected in this study. In addition, 38 matched paracancerous samples were collected. The clinical features were obtained.

Overall survival was defined from surgery date to until the date of last contact. Recurrence-free survival was calculated from the date of surgery until the date of tumour recurrence.

### Cell culture

QBC939 and RBE, two human cholangiocarcinoma cell lines, were cultured in RPMI 1640 medium with 10% foetal bovine serum (HyClone). Primary biliary epithelial cells were bought from ScienCell Research Laboratories (San Diego, CA, USA) and cultured using the media comprising DMEM/F12 (1:1) with 10% foetal bovine serum (FBS), 25 ng/mL epidermal growth factor and 393 ng/mL dexamethasone.

### Cell transfection

GATA6 transfection was performed as previously described [5]. In brief, The CDS template of GATA6 without the miR-124 binding site was synthesized chemically and amplified by PCR. Then, DNA was validated by sequencing and cloned into a pCMX plasmid. A total of 10 μg of GATA6 plasmid (ExGATA6) or empty plasmid (ExControl) were transfected into cells using Lipofectamine LTX and Plus Reagent (Invitrogen, USA).

Cells were transfected with 100 nM miR-124 mimic (ExmiR-124) or 200 nM miR-124 inhibitor (InmiR-124) (Ribobio, Guangzhou, China) in the six-well plate using Lipofectamine 2000 (Invitrogen). After 24 and 48 h, the expression was evaluated by real-time PCR.

### Xenotransplantation of CCA cells into nude mice

CCA cell metastasis was evaluated following xenotransplantation into nude mice by intrasplenic injection as previous described [5]. In brief, Cells (5 × 10^5^) were injected into the spleen of 4-week-old male nude mice (Laboratory Animal Centre, Third Military Medical University). The mice were sacrificed after 1 month. Tumour masses were primarily found in the spleen, liver and bowel grossly at autopsy. All masses except those in the spleen were considered distant metastases. Distant masses were embedded in paraffin, stained with haematoxylin and eosin (HE), and examined under a microscope.

QBC939 cells were transfected with a miR-124 agomir (200 nM) or an negative control for 48 h (200 nM) (Ribobio, Guangzhou, China). Intraperitoneal injection was performed with miR-124 agomir (5 nmol each) or negative control agomir (5 nmol each) twice a week for 2 weeks, which began at week 3 after xenotransplantation.

### Luciferase reporter assays

CCA cells (5 × 10^4^) were seeded in a 48-well plate. The cells were co-transfected with 10 nM miR-124 mimics or NC and 10 ng of firefly luciferase reporter construct. The reporter contained either a wild-type or mutant GATA6 3′-UTR. Luciferase activities were analysed 48 h after transfection using a Dual-Luciferase Reporter Assay System (Promega) in an M200 microplate fluorescence reader (Tecan, Vienna, Austria).

### Cell invasion (Transwell) or migration (wound healing) assay

Cell invasion (Transwell) assay was performed as previously described [5]. Briefly, 2 × 10^5^ cells were suspended in 400 μL of serum-free RPMI 1640 medium and seeded in the top chamber that had been coated with a layer of extracellular matrix (BD Biosciences, USA). Complete medium with serum (500 μl) was added to the bottom chamber. After 48 h of incubation, the cells that had invaded through the extracellular matrix layer to the lower surface of the filters were stained. Photographs of three randomly selected fields of the fixed cells were captured, and cells were counted. Experiments were repeated independently three times.

Cells were seeded in a 6-well plate, grown until confluence, and then starved for 24 h. A linear wound was made by scraping a pipette tip through the confluent cells. The cell motility was measured in terms of wound closure by photographing three random fields 72 h after the wound was made. Experiments were repeated independently three times.

### Real-time PCR

Total RNA extraction was performed using TRIzol reagent (Invitrogen, Grand Island, NY, USA) according to the manufacturer’s instructions. The RNAs were mixed with oligo (dT) or miRNA-specific stem-loop RT primers and reverse transcribed to cDNA using M-MLV Reverse Transcriptase (Promega, Madison, WI, USA). These cDNAs were used to analyse the expression of miR-124 and GATA6 by qPCR using a SYBR premix Ex Taq kit (TaKaRa, Dalian, China). The expression levels were normalized against endogenous β-actin or U6 mRNA as controls. All reactions were performed in an ABI 7500 system (Applied Biosystems, Foster, CA, USA) in triplicate, and the levels of gene expression were calculated using the 2^-ΔΔCT^ method. All primers are shown in Additional file 1: Table S1.

### Western blot analysis

Western blot analysis was performed as previously described [5]. The total protein in CCA cells was isolated using RIPA Lysis Buffer (Beyotime, China). For immunoblotting, equal amounts of proteins were separated on a 5–8% SDS-PAGE gel and electrophoretically transferred onto nitrocellulose membranes (Millipore), which were blocked in TBST containing 5% milk for 2 h at RT and blotted with antibody overnight at 4 °C using anti-GATA6 (1:500, Abcam) or β-actin (1:500, Biotechnology). After washing membranes with TBST and incubating them with either anti-rabbit or anti-mouse horseradish peroxidase-conjugated secondary antibody (Biosynthesis Biotechnology, China) for 2 h at room temperature, immunocomplexes were visualized using chemiluminescence (GE, USA) following the manufacturer’s protocol.

### Statistical analysis

Data were analysed using SPSS 17.0 software. Continuous data were measured with a *t*-test. For categorical data, chi-square analysis or Fisher’s exact test was used. Kaplan-Meier analysis was applied for overall survival and recurrence-free survival. Statistical significance was set at *P* < 0.05.

## Results

### Decreased expression of miR-124 is correlated with higher metastatic behaviour in clinical CCAs

**Fig. 1:**
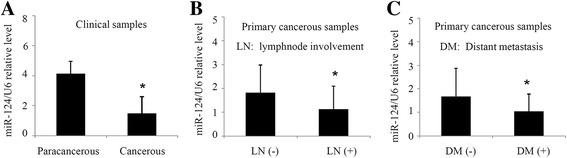
miR-124 expression is correlated with enhanced metastatic behaviour in 57 CCA clinical samples. **a** miR-124 levels between CCA cancerous samples (*N* = 57) and paracancerous samples (*N* = 38). **b** miR-124 levels between CCA primary cancerous samples with lymphnode involvement (*N* = 32) and without lymphnode involvement (*N* = 25). **c** miR-124 levels between CCA primary cancerous samples with distant metastasis (*N* = 17) and without distant metastasis (*N* = 40). *P <0.05

We first compared the miR-124 expression in 57 CCA tissue samples and 38 matched paracancerous samples. The primers used for real time-PCR are in Additional file 1: Table S1. The miR-124 level was significantly lower in cancerous samples compared with paracancerous samples (Fig. 1a), indicating that expression of miR-124 decreased in CCA. Among the 57 cancerous samples, 25 samples with lymph node involvement showed a lower miR-124 level compared with 32 samples without lymph node involvement (Fig. 1b). Moreover, 17 cancerous samples with distant metastasis exhibited a lower miR-124 level compared with 40 samples without distant metastasis (Fig. 1c). miR-124 expression was separated to high and low levels according to the median value. A low miR-124 level significantly related with lymph node involvement but not with gender, age, location, histological grade, or T status (Table 1). The data suggested that miR-124 expression is decreased in CCA, and decreased miR-124 level is correlated with enhanced metastatic behaviour.

**Table 1:** Clinical features of the CCA patients (*n* = 57) according to miR-124 expression grouping

Features	MiR-124 Expression		*P* value^b^
	High (*N* = 29)	Low (*N* = 28)	
Age	55.9 ± 9.7	56.0 ± 10.8	0.969
Gender			0.516
Male	18 (62%)	15 (54%)	
Female	11 (38%)	13 (46%)	
Location			0.682
Intrahepatic	4 (14%)	6 (21%)	
Extrahepatic	25 (86%)	22 (79%)	
Histological Grade			0.580
G1	6 (21%)	4 (14%)	
G2	21 (72%)	20 (71%)	
G3	2 (7%)	4 (14%)	
T classification^a^			0.741
T1	7 (10%)	4 (33%)	
T2	9 (44%)	8 (27%)	
T3	8 (28%)	9 (25%)	
T4	5 (18%)	7 (15%)	
Lymphnode involvement			0.012^c^
Yes	8 (28%)	17 (61%)	
No	21 (72%)	11 (39%)	
Distant metastasis			0.125
Yes	6 (21%)	11 (39%)	
No	23 (79%)	17 (61%)	

The median value was defined as the cut-off point separating high and low expression of miR-124

^a^According to the 6th UICC-TNM staging

^b^ *P* value is for *t* test (continuous variables) or chi-square or Fisher’s exact test (catigorical variables)

^C^ *P* < 0.05, statistical significance

### miR-124 inhibits CCA cell invasion and metastasis in vitro and in vivo

**Fig. 2:**
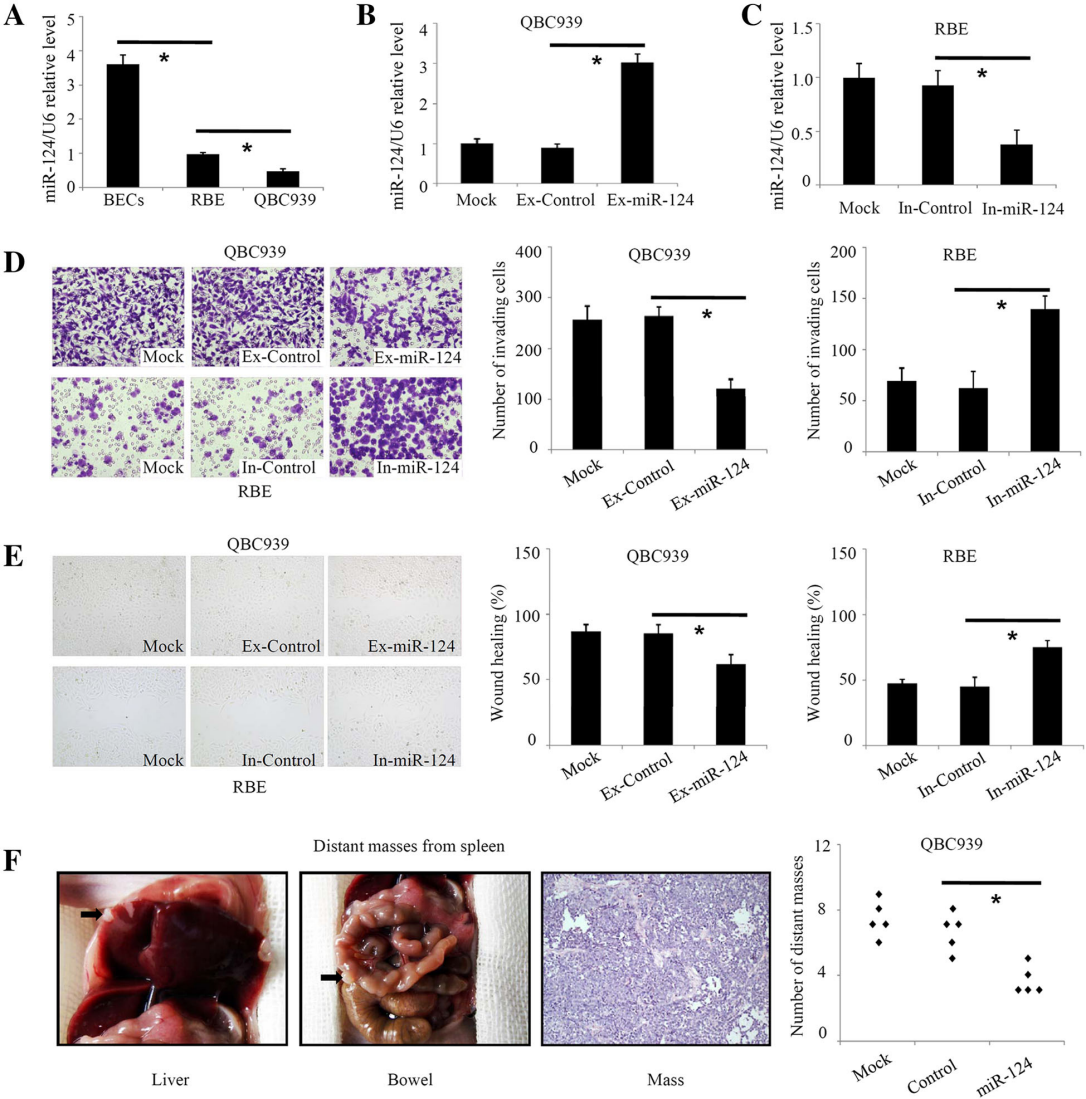
miR-124 inhibits CCA cell invasion and metastasis. **a** The miR-124 levels in two CCA cell lines, QBC939 and RBE, were lower than those in cultured primary biliary epithelial cells, determined by real-time PCR analysis. **b**, **c** Validation of the miR-124 levels after transfection. **d** The effect of miR-124 overexpression on CCA cell invasion, as shown by Transwell assays in vitro. **e** The effect of miR-124 overexpression on CCA cell migration according to wound healing assays in vitro. **f** The effect of miR-124 overexpression on QBC939 cell metastasis following xenotransplantation into nude mice by intrasplenic injection. In-miR-124: CCA cells transfected with miR-124 inhibitors; Ex-miR-124: CCA cells transfected with miR-124 mimics. *P < 0.05

Because the clinical data indicated an negative correlation between miR-124 and metastatic behaviour, we next investigated the effect of miR-124 on migration and invasion in QBC939 and RBE CCA cell lines. Real-time PCR showed the miR-124 levels were decreased in both QBC939 and RBE cells compared with primary biliary epithelial cells (Fig. 2a). Because QBC939 cells exhibited a lower miR-124 level, overexpression of miR-124 was induced in QBC939 cells (Fig. 2b), and downregulation of miR-124 was performed in RBE cells (Fig. 2c). Transwell assays showed a significant decrease in QBC939 cell invasion after miR-124 overexpression (Fig 2d), whereas RBE cell invasion was significantly increased by miR-124 downregulation (Fig. 2d). Moreover, cell migration was significantly decreased by miR-124 overexpression in QBC939 cells and upregulated by miR-124 inhibition in RBE cells (Fig. 2e). These data suggested that miR-124 inhibited CCA migration and invasion in vitro. Next, we investigated the role of miR-124 on CCA cell metastasis. QBC939 cells were injected into the spleens of nude mice. Distant masses were detected at autopsy and by HE staining. The number of distant masses was significantly lower by miR-124 overexpression (Fig. 2f). These data suggested that miR-124 inhibits invasion and metastasis of CCA cells.

### miR-124 downregulates GATA6 expression by directly targeting its 3′-UTR

**Fig. 3:**
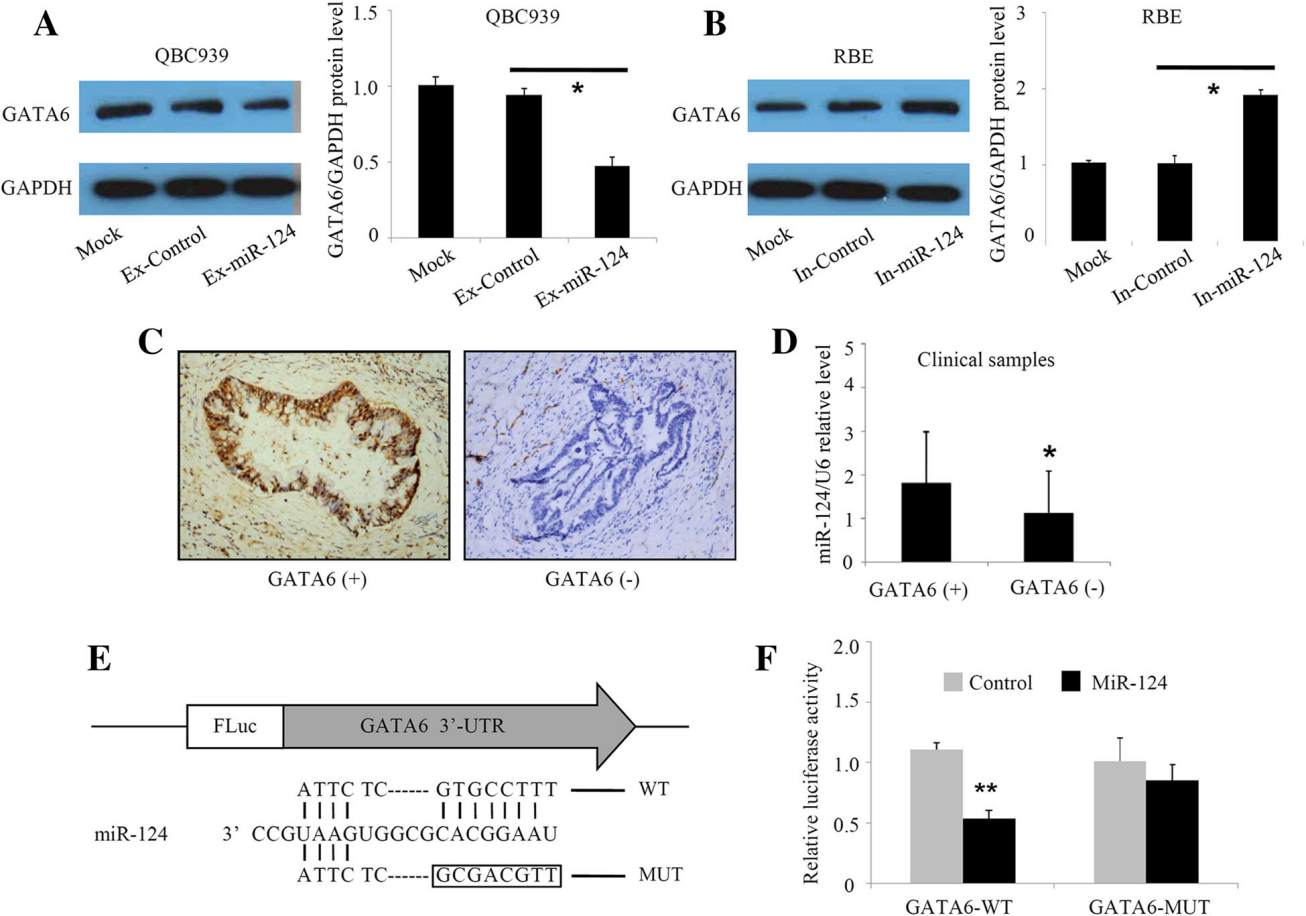
miR-124 downregulates GATA6 expression by directly targeting its 3′-UTR in CCA cells. **a**, **b** The effect of miR-124 overexpression and downregulation on GATA6 protein levels in CCA cells, determined by western blot analysis. **c**, **d** Correlation between GATA6 and miR-124 expression in 57 CCA samples. **e** Mutation of the potential binding site in the 3′-UTR of GATA6. **f** The effect of miR-124 on the relative luciferase activity of the GATA6 wild-type and mutant 3′-UTR constructs. In-miR-124: CCA cells transfected with miR-124 inhibitors; Ex-miR-124: CCA cells transfected with miR-124 mimics. *P < 0.05, **P < 0.01

We then investigated the role of miR-124 on GATA6 expression in CCA cells. Western blot analysis showed that GATA6 protein levels were downregulated by miR-124 overexpression in QBC939 cells (Fig. 3a) and upregulated by miR-124 inhibition in RBE cells (Fig. 3b). The correlation between miR-124 and GATA6 was also evaluated in the 57 clinical CCA samples. GATA6 protein expression was observed using immunohistochemistry (IHC). A total of 27 samples (47%) showed GATA6-positive staining (Fig. 3c). miR-124 levels was negatively correlated with GATA6 in 57 CCA samples (Fig. 3d). The above data indicated that GATA6 might be regulated by miR-124 in CCA cells. miRNAs perform post-transcriptional regulation through binding to the mRNA 3′-UTR. Because a bioinformatics prediction analysis indicated that the 3′-UTR of GATA6 mRNA had a target site for miR-124, we determined whether miR-124 downregulated GATA6 by targeting its 3′-UTR. The luciferase reporters were constructed containing mutant or wild-type 3′-UTRs of the GATA6 gene (Fig. 3e) and cotransfected them with miR-124 mimics into QBC939 cells. miR-124 significantly inhibited the luciferase activity of reporter genes containing the wild-type GATA6 3′-UTR, whereas the inhibition was significantly rescued by mutating the miR-124-binding site (Fig. 3f). Taken together, our data indicate that miR-124 downregulates GATA6 expression by directly targeting its 3′-UTR.

### miR-124 inhibits CCA invasion and metastasis by downregulating GATA6

**Fig. 4:**
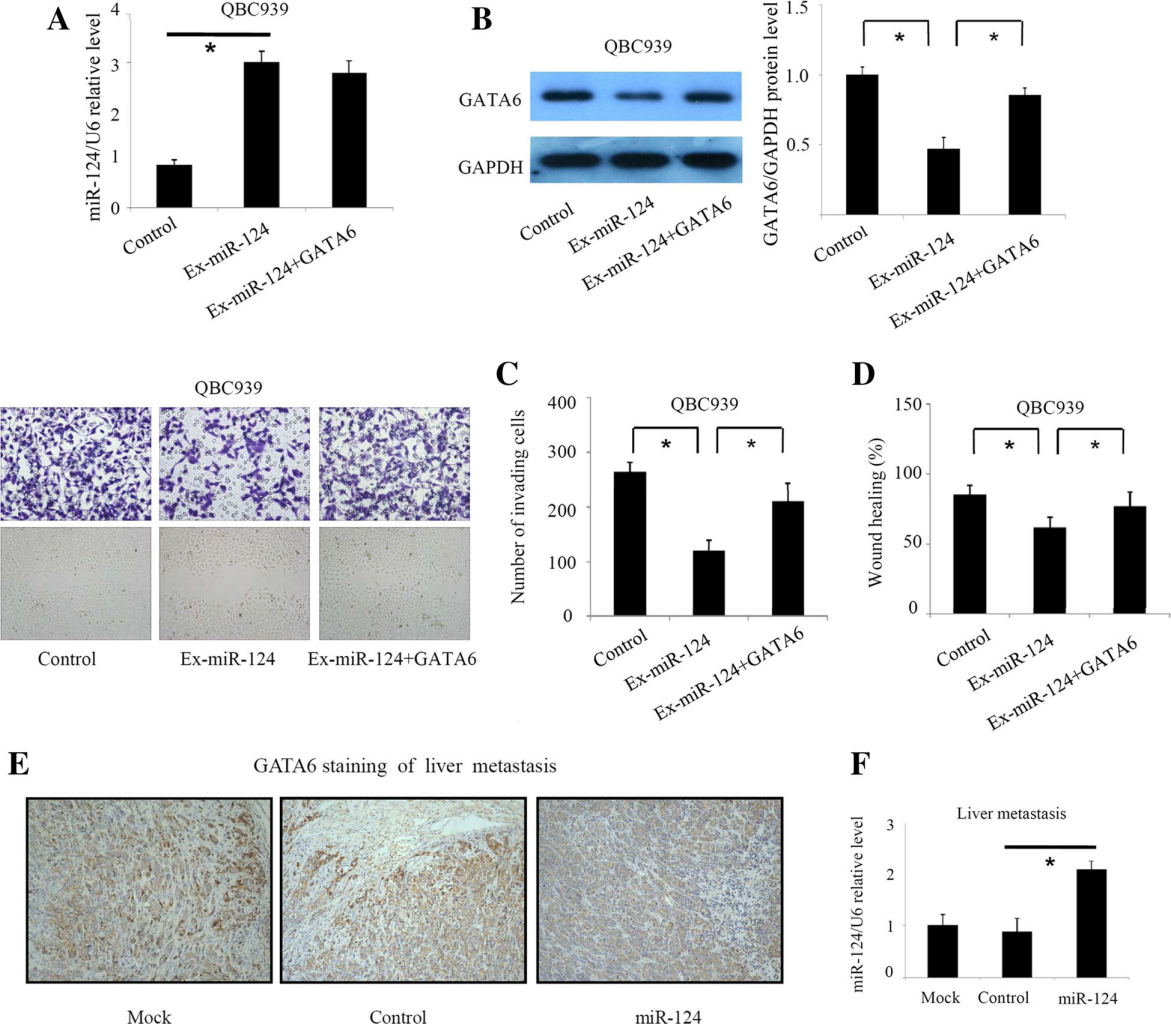
miR-124 inhibits CCA cell invasion and metastasis by downregulating GATA6. **a**, **b** Validation of miR-124 and GATA6 levels after transfection. **c**, **d** Remedial expression of GATA6 significantly abrogated the miR-124-induced suppression of QBC939 cell invasion and migration in Transwell and wound healing assays. **e** IHC analysis of GATA6 expression in the liver masses of xenotransplanted mice. **f** Validation of miR-124 expression in the liver masses. Ex-miR-124: CCA cells transfected with miR-124 mimics. *P < 0.05

To investigate whether miR-124 inhibited CCA invasion and metastasis through targeting GATA6, plasmids carrying the entire coding sequence of GATA6 without the miR-124 binding sites were transfected into QBC939 cells to rescue miR-124 -induced downregulation of GATA6 (Fig. 4a and b). Remedial GATA6 expression significantly abrogated the miR-124-induced suppression of QBC939 cell invasion and migration in Transwell and wound healing assays (Fig. 4c and d). In addition, we detected GATA6 expression in liver masses from the xenotransplanted mice using IHC. GATA6 was decreased in the miR-124 group (Fig. 4e), which was consistent with the diminished metastatic behaviour. We also determined the miR-124 expression level in the liver masses and found that miR-124 was increased in the miR-124 group (Fig. 4f). Our data suggest that miR-124 might inhibit invasion and metastasis of CCA cells through downregulation of GATA6.

### miR-124 expression is negatively correlated with prognosis in CCA patients

**Fig. 5:**
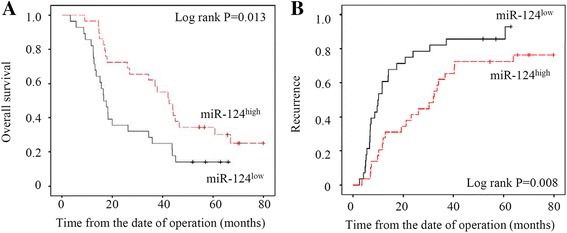
Expression of miR-124 affected overall survival (**a**) and recurrence (**b**) in 57 CCA patients following surgical resection, determined by Kaplan–Meier analysis

Because recurrence and metastasis are major factors for a poor prognosis, we next investigated the relation between the expression of miR-124 and prognosis in 57 CCA patients. miR-124 expression were separated to high and low levels by the median value. We compared postoperative and disease-free survival between miR-124^high^ and miR-124^low^ patients with a Kaplan-Meier analysis. MiR-124^low^ patients had significantly poorer overall survival (Fig. 5a) and a higher rate of recurrence (Fig. 5b). Therefore, the result suggest that miR-124 expression is negatively correlated with the prognosis of CCA patients.

## Discussion

Deregulation of miRs has been reported in various cancer tissues from numerous profiling data [17]. Moreover, these small non-coding RNAs can function as oncogenes or tumour-suppressor genes that contribute to tumourigenesis and progression [8]. In the present study, we found miR-124 was downregulated in cancerous samples from CCA patients. Downregulation of miR-124 contributed to CCA invasion and metastasis, indicating its tumour-suppressor function. In addition, we demonstrated that GATA6 is a new potential target of miR-124. miR-124 downregulated GATA6 by targeting the 3′-UTR. Moreover, miR-124 inhibited CCA cell invasion and metastasis by downregulating GATA6. Taken together, these findings suggest an important role of miR-124 in CCA invasive and metastatic potential.

miR-124 has been reported to be downregulated by the hepatitis C virus (HCV) core protein in HCV-related intrahepatic CCA [18]. Here, we found that miR-124 was downregulated in both intrahepatic and extrahepatic CCA. Furthermore, many miR-124-decreased CCA patients did not have HCV infections, indicating that there might be other mechanisms regulating miR-124 expression during CCA progression. Recent studies have shown that aberrant DNA methylation plays important roles in miR-124 downregulation in several types of cancers, including hepatocellular carcinoma, gastric cancer, colon cancer, lung cancer, lymphoma, and pancreatic cancer [12, 19–21]. Whether DNA methylation also participates in the downregulation of miR-124 in CCA, especially HCV-negative CCA, needs to be investigated further.

Dysregulation of GATA6 has been reported in several types of cancers. It functions as a promoter and suppressor according to the tumour origin. Our previous study showed that GATA6 is aberrantly upregulated in CCA, which indicates that it functions as a potential oncogene [5]. Kwei et al. [6] reported that an increased gene copy number contributes to the aberrant expression of GATA6. Wang et al. [22] reported that DeltaNp63alpha, the oncogenic isoform of the p63 protein, regulates GATA6 expression in gastric cancer. Here, we showed that a downregulation of miR-124 expression might contribute to the aberrant expression of GATA6 in CCA. Both of the above studies suggest that the dysregulation of GATA6 expression during CCA progression is controlled by multiple pathways, including the enhancement of positive regulation pathways and the inhibition of negative regulation pathways.

GATA6 participates in cancer progression by regulating certain key molecules. Among these key molecules, 67LR is an important protein that is regulated by GATA6 through binding to its prompter region in CCA [5]. 67LR is a cell surface non-integrin receptor expressed in epithelial cells that mediates cell attachment to laminin. An increase in 67LR expression has been found in a variety of cancers including CCA [23, 24]. 67LR promotes CCA cell invasion and metastasis through various mechanisms, including facilitating tumour cell invasion into adjacent tissue by upregulating proteolytic enzymes [25, 26] and assisting tumour cell survival in the circulatory system by promoting immune privilege [27]. By targeting GATA6, miR-124 may also downregulate 67LR expression and play important roles in these processes during CCA cell metastasis. The present results, together with the above evidence, indicate the important role of the miR-124-GATA6-67LR pathway in CCA cell invasion and metastasis.

Although our results showed a potential new target of miR-124 and the important role of this pathway in CCA cell metastasis, there must be other pathways by which miR-124 exerts its effects. Previous studies have shown that miR-124 inhibits cancer cell invasion and metastasis by targeting other molecules, including slug [13], Rac-1 [12, 28], SMYD3 [18], SphK1 [29], and ROCK1 [30]. GATA6 is also reported to participate in cancer cell invasion and metastasis by regulating other proteins, such as urokinase plasminogen activator [31], slug [32] and BMP4 [33]. All of this evidence suggests that invasion and metastasis of cancers including CCA is regulated by a molecular network. Our previous and present results suggest that miR-124 and GATA6 play important roles in this network.

In addition to metastasis, GATA6 is reported to play important roles in tumourigenesis, self-renewal of cancer stem cells, and proliferation and apoptosis of various types of cancer [22, 33–35]. miR-124 is also reported to participate in regulating angiogenesis, chemosensitivity and proliferation of cancers [14, 36, 37]. Whether miR-124 participates in regulating other hallmarks of CCA by targeting GATA6 needs to be investigated further.

## Conclusion

In conclusion, we show miR-124 is downregulated in CCA. miR-124 decreases GATA6 expression by directly targeting its 3′-UTR, which in turn inhibits CCA cell invasion and metastasis. We therefore propose that the restoration of miR-124 expression might represent a potential strategy for CCA therapy.

## Additional file

Additional file 1: Table S1. Primer sequences used in real-time PCR. (DOC 56 kb)

## Abbreviations

3′-UTR: Untranslated regions
CCA: Cholangiocarcinoma
GATA6: GATA binding protein 6

## References

[1] Friman S (2011). Cholangiocarcinoma--current treatment options. Scand J Surg.

[2] Valastyan S, Weinberg RA (2011). Tumor metastasis: molecular insights and evolving paradigms. Cell.

[3] Rizvi S, Gores GJ (2013). Pathogenesis, diagnosis, and management of cholangiocarcinoma. Gastroenterology.

[4] Maemura K, Natsugoe S, Takao S (2014). Molecular mechanism of cholangiocarcinoma carcinogenesis. J Hepatobiliary Pancreat Sci.

[5] Tian F, Li D, Chen J, Liu W, Cai L, Li J (2013). Aberrant expression of GATA binding protein 6 correlates with poor prognosis and promotes metastasis in cholangiocarcinoma. Eur J Cancer.

[6] Kwei KA, Bashyam MD, Kao J, Ratheesh R, Reddy EC, Kim YH (2008). Genomic profiling identifies GATA6 as a candidate oncogene amplified in pancreatobiliary cancer. PLoS Genet.

[7] Iorio MV, Croce CM (2012). microRNA involvement in human cancer. Carcinogenesis.

[8] Chen CZ (2005). MicroRNAs as oncogenes and tumor suppressors. N Engl J Med.

[9] Esquela-Kerscher A, Slack FJ (2006). Oncomirs - microRNAs with a role in cancer. Nat Rev Cancer.

[10] Bouyssou JM, Manier S, Huynh D, Issa S, Roccaro AM, Ghobrial IM (2014). Regulation of microRNAs in cancer metastasis. Biochim Biophys Acta.

[11] Zheng F, Liao YJ, Cai MY, Liu YH, Liu TH, Chen SP (2012). The putative tumour suppressor microRNA-124 modulates hepatocellular carcinoma cell aggressiveness by repressing ROCK2 and EZH2. Gut.

[12] Wang P, Chen L, Zhang J, Chen H, Fan J, Wang K (2013). Methylation-mediated silencing of the miR-124 genes facilitates pancreatic cancer progression and metastasis by targeting Rac1. Oncogene.

[13] Liang YJ, Wang QY, Zhou CX, Yin QQ, He M, Yu XT (2013). MiR-124 targets slug to regulate epithelial-mesenchymal transition and metastasis of breast cancer. Carcinogenesis.

[14] Kang S, Zhao Y, Hu K, Xu C, Wang L, Liu J (2014). miR-124 exhibits antiproliferative and antiaggressive effects on prostate cancer cells through PACE4 pathway. Prostate.

[15] Li Z, Wang X, Li W, Wu L, Chang L, Chen H (2016). miRNA-124 modulates lung carcinoma cell migration and invasion. Int J Clin Pharmacol Ther.

[16] Cai JJ, Qi ZX, Chen LC, Yao Y, Gong Y, Mao Y (2016). miR-124 suppresses the migration and invasion of glioma cells in vitro via Capn4. Oncol Rep.

[17] Iorio MV, Croce CM (2012). MicroRNA dysregulation in cancer: diagnostics, monitoring and therapeutics. A comprehensive review. EMBO Mol Med.

[18] Zeng B, Li Z, Chen R, Guo N, Zhou J, Zhou Q (2012). Epigenetic regulation of miR-124 by hepatitis C virus core protein promotes migration and invasion of intrahepatic cholangiocarcinoma cells by targeting SMYD3. FEBS Lett.

[19] Furuta M, Kozaki KI, Tanaka S, Arii S, Imoto I, Inazawa J (2010). miR-124 and miR-203 are epigenetically silenced tumor-suppressive microRNAs in hepatocellular carcinoma. Carcinogenesis.

[20] Lujambio A, Ropero S, Ballestar E, Fraga MF, Cerrato C, Setien F (2007). Genetic unmasking of an epigenetically silenced microRNA in human cancer cells. Cancer Res.

[21] Ando T, Yoshida T, Enomoto S, Asada K, Tatematsu M, Ichinose M (2009). DNA methylation of microRNA genes in gastric mucosae of gastric cancer patients: its possible involvement in the formation of epigenetic field defect. Int J Cancer.

[22] Wang H, Liu Z, Li J, Zhao X, Wang Z, Xu H (2012). DeltaNp63alpha mediates proliferation and apoptosis in human gastric cancer cells by the regulation of GATA-6. Neoplasma.

[23] Nelson J, McFerran NV, Pivato G, Chambers E, Doherty C, Steele D (2008). The 67 kDa laminin receptor: structure, function and role in disease. Biosci Rep.

[24] Li D, Chen J, Gao Z, Li X, Yan X, Xiong Y (2009). 67-kDa laminin receptor in human bile duct carcinoma. Eur Surg Res.

[25] Givant-Horwitz V, Davidson B, Reich R (2004). Laminin-induced signaling in tumor cells: the role of the M(r) 67,000 laminin receptor. Cancer Res.

[26] Liu L, Sun L, Zhao P, Yao L, Jin H, Liang S (2010). Hypoxia promotes metastasis in human gastric cancer by up-regulating the 67-kDa laminin receptor. Cancer Sci.

[27] Duan SG, Cheng L, Li DJ, Zhu J, Xiong Y, Li XW (2010). The role of MAPK-ERK pathway in 67-kDa laminin receptor-induced FasL expression in human cholangiocarcinoma cells. Dig Dis Sci.

[28] Zhang H, Wang Q, Zhao Q, Di W (2013). MiR-124 inhibits the migration and invasion of ovarian cancer cells by targeting SphK1. J Ovarian Res.

[29] Geng S, Zhang X, Chen J, Liu X, Zhang H, Xu X (2014). The tumor suppressor role of miR-124 in osteosarcoma. PLoS One.

[30] An L, Liu Y, Wu A, Guan Y (2013). microRNA-124 inhibits migration and invasion by down-regulating ROCK1 in glioma. PLoS One.

[31] Belaguli NS, Aftab M, Rigi M, Zhang M, Albo D, Berger DH (2010). GATA6 promotes colon cancer cell invasion by regulating urokinase plasminogen activator gene expression. Neoplasia.

[32] Song Y, Tian T, Fu X, Wang W, Li S, Shi T (2015). GATA6 is overexpressed in breast cancer and promotes breast cancer cell epithelial-mesenchymal transition by upregulating slug expression. Exp Mol Pathol.

[33] Whissell G, Montagni E, Martinelli P, Hernando-Momblona X, Sevillano M, Jung P (2014). The transcription factor GATA6 enables self-renewal of colon adenoma stem cells by repressing BMP gene expression. Nat Cell Biol.

[34] Tsuji S, Kawasaki Y, Furukawa S, Taniue K, Hayashi T, Okuno M (2014). The miR-363-GATA6-Lgr5 pathway is critical for colorectal tumourigenesis. Nat Commun.

[35] Lin L, Bass AJ, Lockwood WW, Wang Z, Silvers AL, Thomas DG (2012). Activation of GATA binding protein 6 (GATA6) sustains oncogenic lineage-survival in esophageal adenocarcinoma. Proc Natl Acad Sci U S A.

[36] Shi Z, Chen Q, Li C, Wang L, Qian X, Jiang C (2014). MiR-124 governs glioma growth and angiogenesis and enhances chemosensitivity by targeting R-Ras and N-Ras. Neuro Oncol.

[37] Park SY, Kim H, Yoon S, Bae JA, Choi SY, Jung YD (2014). KITENIN-targeting microRNA-124 suppresses colorectal cancer cell motility and tumorigenesis. Mol Ther.

