# Supplementary material for: miR-124 targets GATA6 to suppress cholangiocarcinoma cell invasion and metastasis
Source: BMC Cancer. 2017 Mar 7;17:175. doi: 10.1186/s12885-017-3166-z (PMC5339982; doi:10.1186/s12885-017-3166-z)
Supplement: Additional file 1: Table S1. — Primer sequences used in real-time PCR. (DOC 56 kb) [file 12885_2017_3166_MOESM1_ESM.doc]

**Table S1 Primer sequence used in real-time PCR.**

| Name | Sequence (5’-3’) | Amplicon (bp) |
| --- | --- | --- |
| miR-124 | Forward: GATACTCATAAGGCACGCGG | 64 |
| miR-124 | Reverse: GTGCAGGGTCCGAGGT |  |
| U6 | Forward: CGCTTCGGCAGCACATATAC | 60 |
| U6 | Reverse: CAGGGGCCATGCTAATCTT |
| GATA6 | Forward: CCAACTTCCACCTCTTCTAAC | 150 |
|  | Reverse: TTGACCCGAATACTTGAGC |  |
| β-actin | Forward: CCATGTACGTTGCTATCCAGG | 252 |
|  | Reverse: TCTCCTTAATGTCACGCACGA |  |
